# The Gibraltar Epidemic

**Published:** 1829-04-01

**Authors:** 


GIBRALTAR EPIDEMIC.

A private letter from a friend at Gibraltar informs us that a commission of distinguished physicians, appointed by the French government, have been for some time in active operation at Gibraltar, in the investigation of the late epidemic. To this commission Sir George Don has added Dr. DAVID BARRY, so well and so favourably known in this country, by many ingenious works and memoirs. The French commission includes M. Chervin, M. Louis, (the distinguished author of the work on phthisis) and M. Trousseau. For some days after their arrival on the rock, the commissioners worked hard in the dissecting-rooms, and are now employed in tracing the progress of the disease from house to house—from individual to individual, accompanied by the local authorities. They take down the depositions of persons, as to whether they had passed the fever in any previous epidemic—whether they had it this year—the symptoms—the communications they may have had with other places or persons, &c. &c. &c. The medical topography of Gibraltar is also carefully investigated, as well as the other diseases which reigned at the time, or preceded the epidemic fever. All these facts, histories, and circumstances are faithfully noted, and regularly signed by the different witnesses—and from these it is intended to draw up a series of statistical and sanatory conclusions, excluding all theory and speculation.

It is evident that this is the way to get at truth; and we cannot but admire the wisdom and liberality of the French government in thus taking cognizance of matters of science and health, in which they are but very remotely concerned. No government can be half so much interested in the above investigation as our own—and yet, had the French commission not proceeded to Gibraltar, not a single atom of inquiry would ever have been instituted by our own government. Even as it is, not a farthing of expense is occurred by the British government. The Governor of Gibraltar merely permits (rather than commands) an English physician to be added to the commission of investigation! We shall look with much anxiety to the result of this inquiry, and shall not fail to lay the particulars before our readers.

---

LIII.

